# Ethyl 2-[(2-oxo-2*H*-chromen-6-yl)­oxy]acetate

**DOI:** 10.1107/S2056989024004729

**Published:** 2024-05-31

**Authors:** Navneet Goyal, James P. Donahue, Anthony Thompson, Xiaodong Zhang, Joel T. Mague, Maryam Foroozesh

**Affiliations:** a Department of Chemistry, Xavier University of Louisiana, 1 Drexel Dr., New Orleans, Louisiana 70125, USA; b Department of Chemistry, Tulane University, 6400 Freret Street, New Orleans, Louisiana 70118-5698, USA; Texas A & M University, USA

**Keywords:** chromen-2-one, coumarin, weak C—H⋯O H-bonding, sheet structure, π–π stacking, crystal structure

## Abstract

Ethyl 2-[(2-oxo-2*H*-chromen-6-yl)­oxy]acetate, C_13_H_12_O_5_, a member of the pharmacologically important class of coumarins, crystallizes in the monoclinic *C*2/*c* space group in the form of sheets, within which mol­ecules are related by inversion centers and 2_1_ axes. Multiple C—H⋯O weak hydrogen-bonding inter­actions reinforce this pattern. The planes of these sheets are oriented in the approximate direction of the *ac* face diagonal. Inter­sheet inter­actions are a combination of coumarin system π–π stacking and additional C—H⋯O weak hydrogen bonds between ethyl acet­oxy groups.

## Abstract 2

Ethyl 2-[(2-oxo-2*H*-chromen-6-yl)­oxy]acetate, a coumarin derivative, crystallizes in sheets, within which mol­ecules are held by weak C—H⋯O hydrogen-bonding inter­actions and between which mol­ecules inter­act by π–π stacking and additional C—H⋯O weak hydrogen bonds between ethyl acet­oxy groups.

## 1. Chemical context

Chromen-2-one, also known as coumarin, and its derivatives hold considerable significance in both natural product and synthetic organic chemistry. Coumarin’s structure is characterized by a benzene ring fused to an α-pyrone ring, which makes it valuable in pharmaceutical research (Murray *et al.*, 1982 ▸). Coumarin derivatives have shown biological activity as anti­cancer (Emami & Dadashpour, 2015 ▸), anti­oxidant (Matos *et al.*, 2017 ▸), anti­coagulant (Satish, 2016 ▸) and anti­neuro­degenerative agents (Jameel *et al.*, 2016 ▸). We have previously reported a number of synthetically derived mol­ecules based on coumarin, chromene and flavone as substrates/inhibitors of several important cytochrome P450 enzymes, including P450s 1A1, 1A2, and 2A6 (Goyal *et al.*, 2023 ▸; Foroozesh *et al.*, 1997 ▸). As part of an ongoing program of research into the pharmacological properties of coumarin derivatives, we have undertaken the synthesis of ethyl 2-[(2-oxo-2*H*-chromen-6-yl)­oxy]acetate, the structural characterization of which we report herein. 
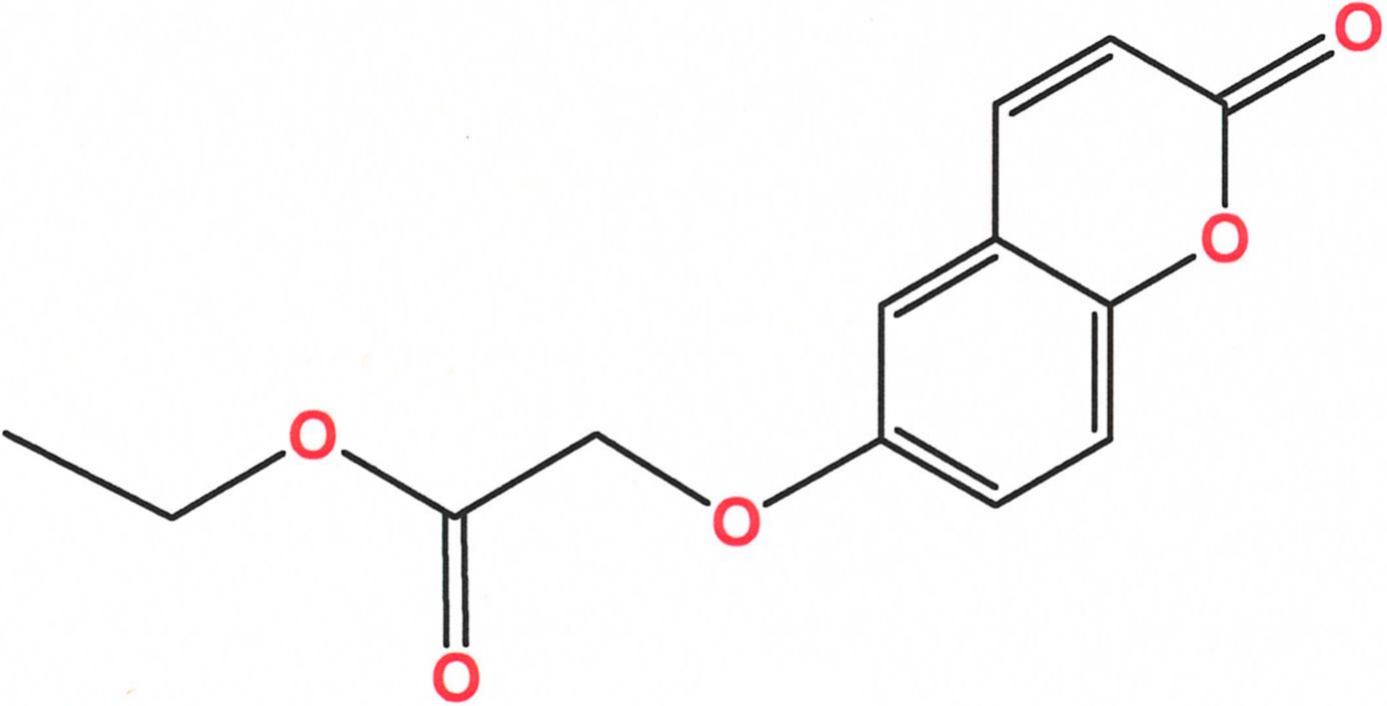


## 2. Structural commentary

**Figure 1:**
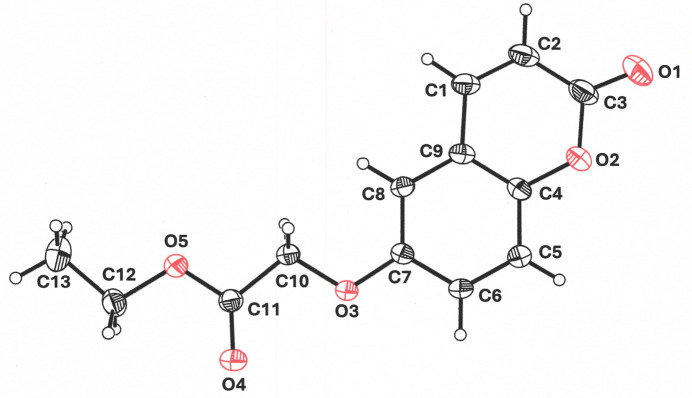
Displacement ellipsoid plot of ethyl 2-[(2-oxo-2*H*-chromen-6-yl)­oxy]acetate with complete labeling of non-hydrogen atoms. Ellipsoids are shown at the 50% probability level.

Ethyl 2-[(2-oxo-2*H*-chromen-6-yl)­oxy]acetate deposits in the form of colorless blocks by slow cooling of a 2:1 ethyl acetate:hexa­nes solution. The mol­ecule crystallizes in completely ordered fashion with the appended ethyl oxyacetate group at the 6-position arranged in a fully extended, linear arrangement (Fig. 1 ▸). Thus, all non-hydrogen atoms of the mol­ecule reside within the same plane with an average deviation of 0.0457 Å.

**Figure 2:**
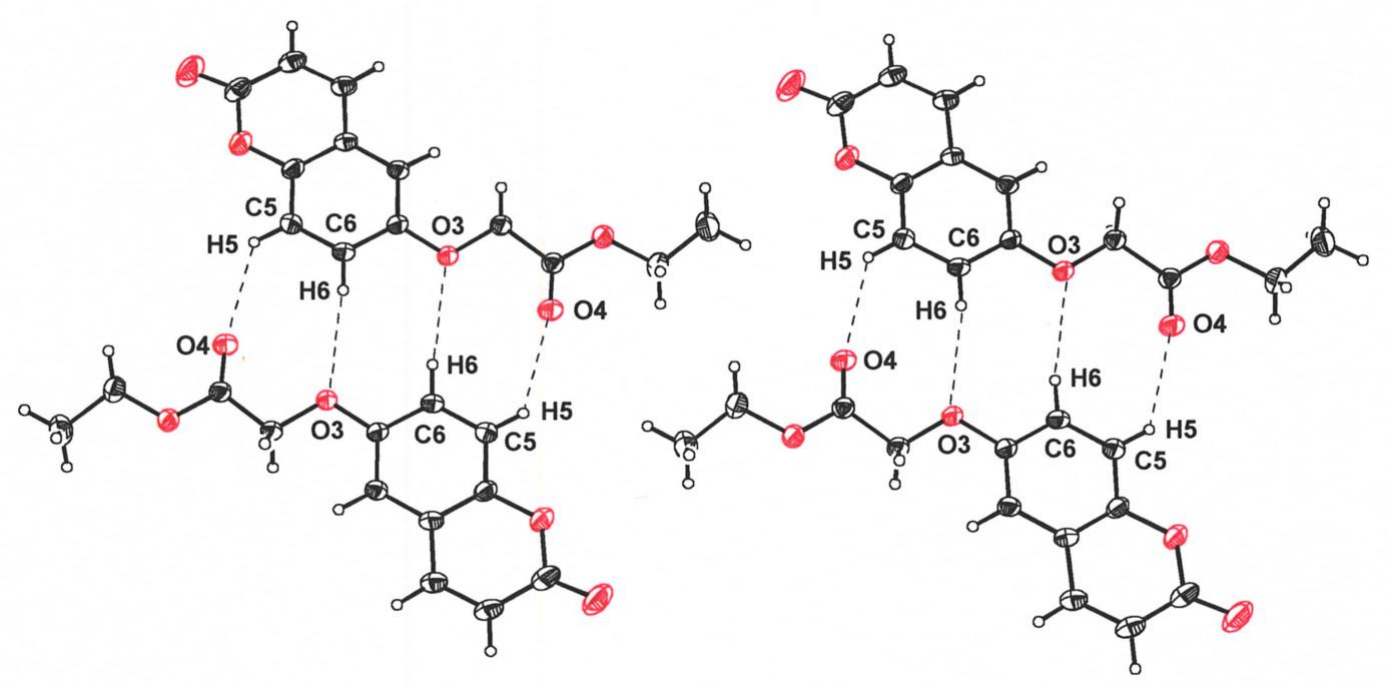
Planar centrosymmetric dyads of ethyl 2-[(2-oxo-2*H*-chromen-6-yl)­oxy]acetate showing the C—H⋯O weak inter­actions that guide the packing arrangement. The H5⋯O4 and H6⋯O3 distances are 2.47 and 2.65 Å, respectively. The symmetry transformation relating mol­ecules through these hydrogen bonds is −*x* + 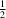 , −*y* + 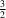 , −*z* + 1. Displacement ellipsoids are presented at the 50% probability level.

**Figure 3:**
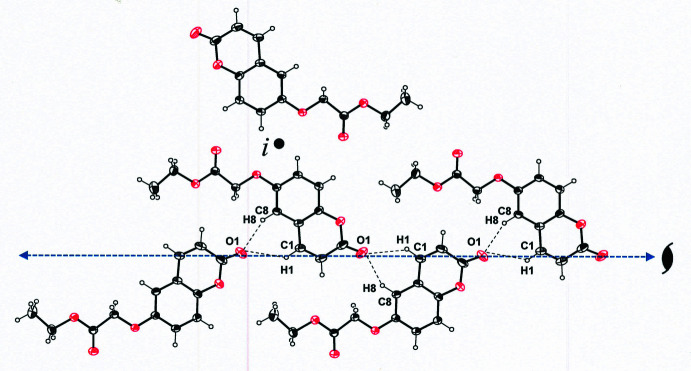
Rows of ethyl 2-[(2-oxo-2*H*-chromen-6-yl)­oxy]acetate mol­ecules to both sides of, and in the same plane as, the centrosymmetric diads in Fig. 2 ▸. These mol­ecules are related to those in the centrosymmetric dyads by a 2_1_ operation, the position for one such axis being shown. This patterned arrangement is assisted by C8—H8⋯O1 and by C1—H1⋯O1 close contacts, in which the corresponding H8⋯O1 and H1⋯O1 distances are 2.27 and 2.61 Å. The symmetry transformation whereby one mol­ecule is converted to the other across these hydrogen bonds is −*x* + 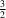 , *y* − 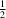 , −*z* + 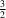 . Ellipsoids are shown at the 50% probability level.

**Figure 4:**
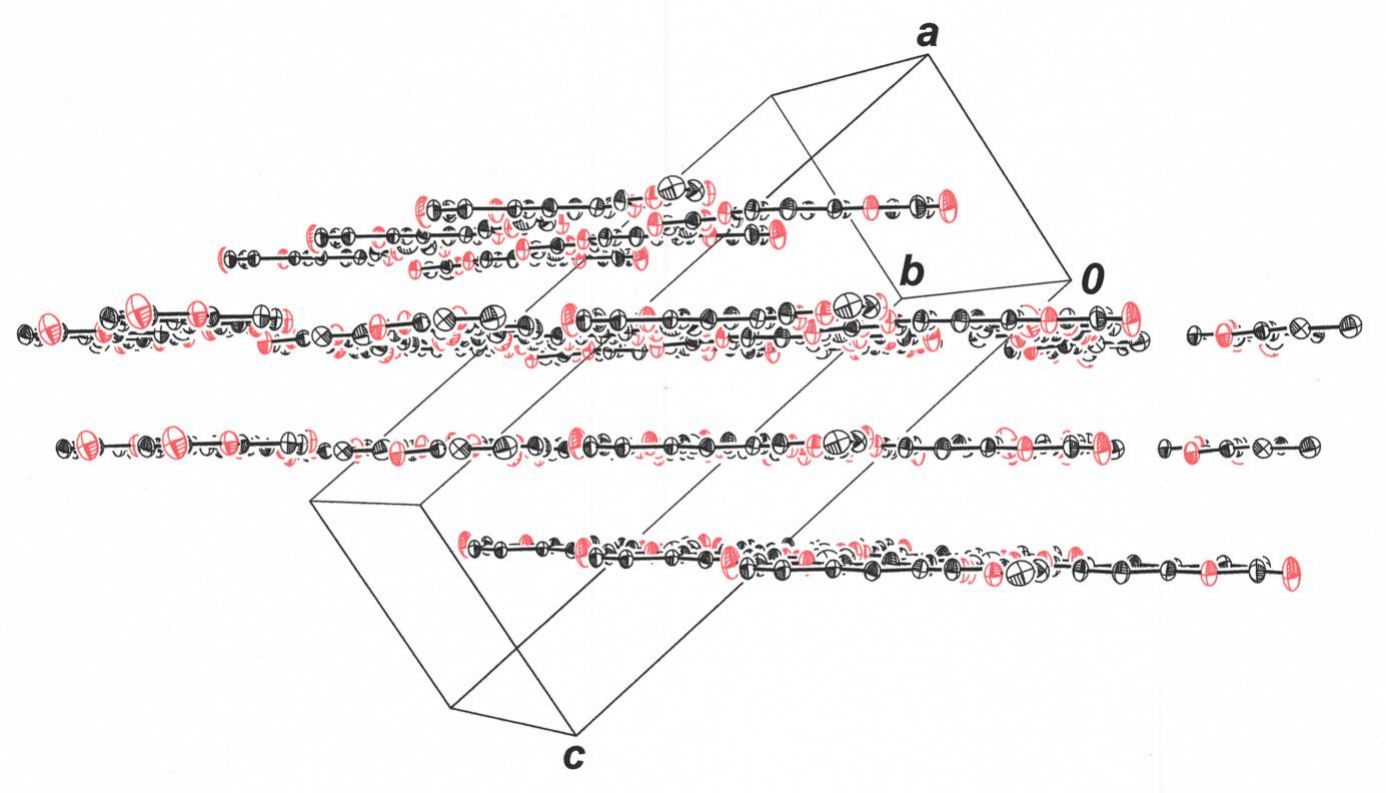
Packing diagram for ethyl 2-[(2-oxo-2*H*-chromen-6-yl)­oxy]acetate illustrating the arrangement of mol­ecules into sheets in the approximate direction of the *ac* face diagonal of the unit cell. All H atoms are omitted for clarity, and displacement ellipsoids are drawn at 50% probability.

**Figure 5:**
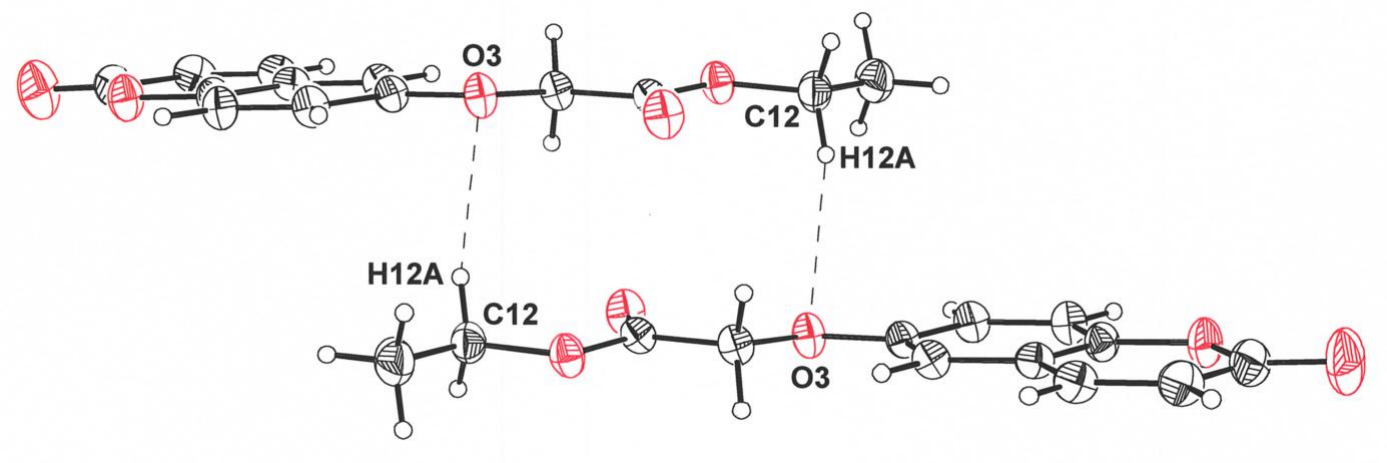
Weak C—H⋯O inter­actions between centrosymmetric pairs of mol­ecules of ethyl 2-[(2-oxo-2*H*-chromen-6-yl)­oxy]acetate in different sheets. The H12*A*⋯O3 distance is 2.57 Å, and the symmetry transformation relating these mol­ecules is −*x* + 1, −*y* + 1, −*z* + 1. Displacement ellipsoids are presented at the 50% probability level.

Coplanar pairs of ethyl 2-[(2-oxo-2*H*-chromen-6-yl)­oxy]acetate mol­ecules are organized in a head-to-tail fashion by apparent C5—H5⋯O4 and C6—H6⋯O3 weak hydrogen-bonding inter­actions around an inversion center (Table 1 ▸, Fig. 2 ▸). The adjoining rows of mol­ecules above and below those shown in Fig. 2 ▸ are related by 2_1_ screw axes to those in these centrosymmetric dyads, with which they form C8—H8⋯O1 and C1—H1⋯O1 hydrogen bonds (Fig. 3 ▸). The replication of these rows of mol­ecules, which are alternately related by inversion centers and 2_1_ axes, creates sheets whose planes lie approximately in the direction of the *ac* face diagonal of the unit cell (Fig. 4 ▸). Mol­ecules between sheets are also related by inversion centers (Fig. 5 ▸) and enjoy pairs of C12—H12*A*⋯O3 hydrogen-bond contacts. The layered packing arrangement is guided by π–π stacking between the coumarin ring systems, with a separation of 3.4460 (6) Å between the centroids of the α-pyrone rings (C1–C3/O2/C4/C9) of adjacent mol­ecules, as assessed by *PLATON* (Spek, 2020 ▸). This distance is only modestly greater than the 3.35 Å separation between the sheets of carbon atoms in graphite (Chung, 2002 ▸) and is reinforced by the hydrogen bonding between extended ethyl oxyacetate chains in adjacent layers (Fig. 5 ▸).

**Figure 6:**
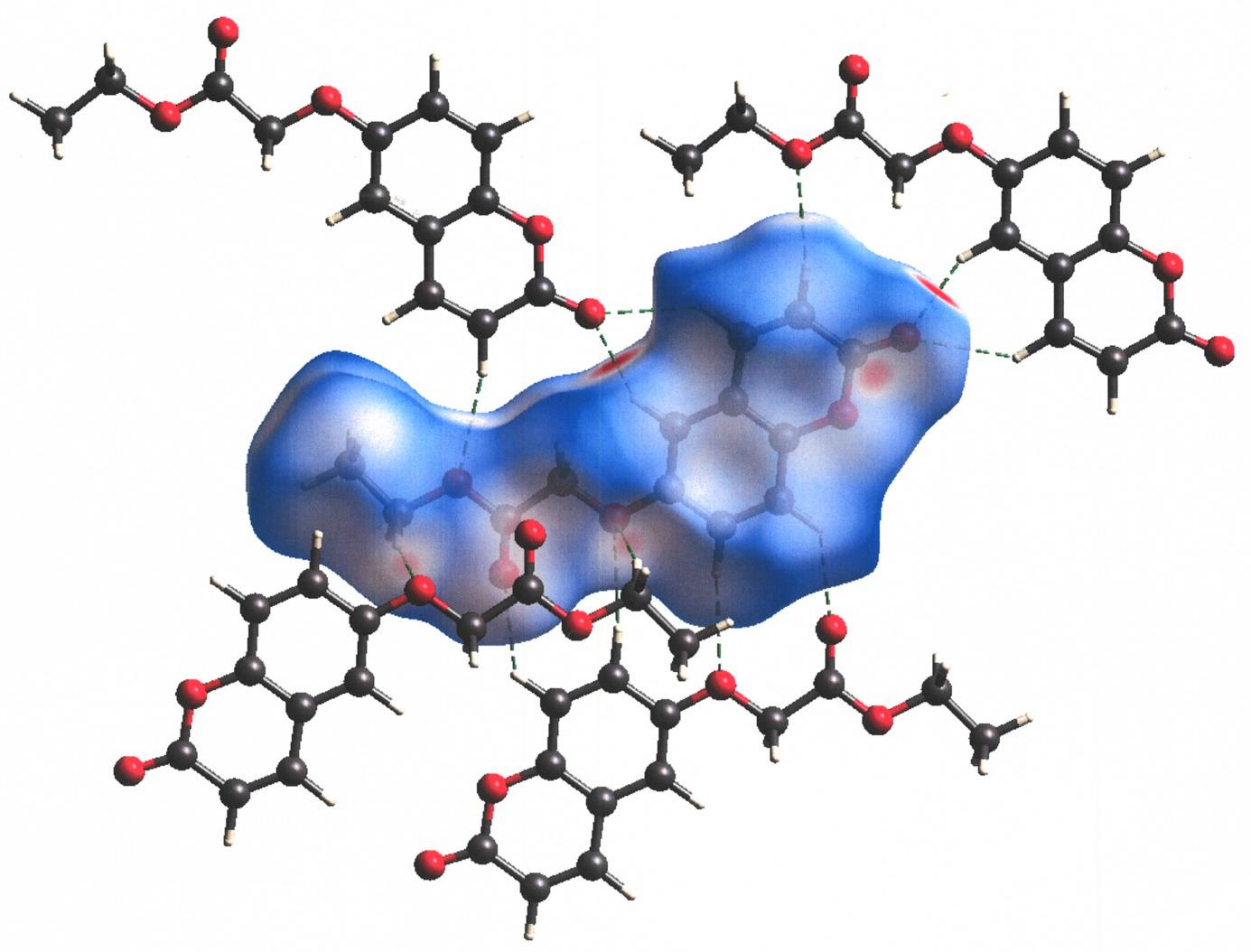
Hirshfeld surface for ethyl 2-[(2-oxo-2*H*-chromen-6-yl)­oxy]acetate with *d*_norm_ set between −0.3446 and 1.3365. Close inter­molecular contacts are depicted with dashed lines.

**Figure 7:**
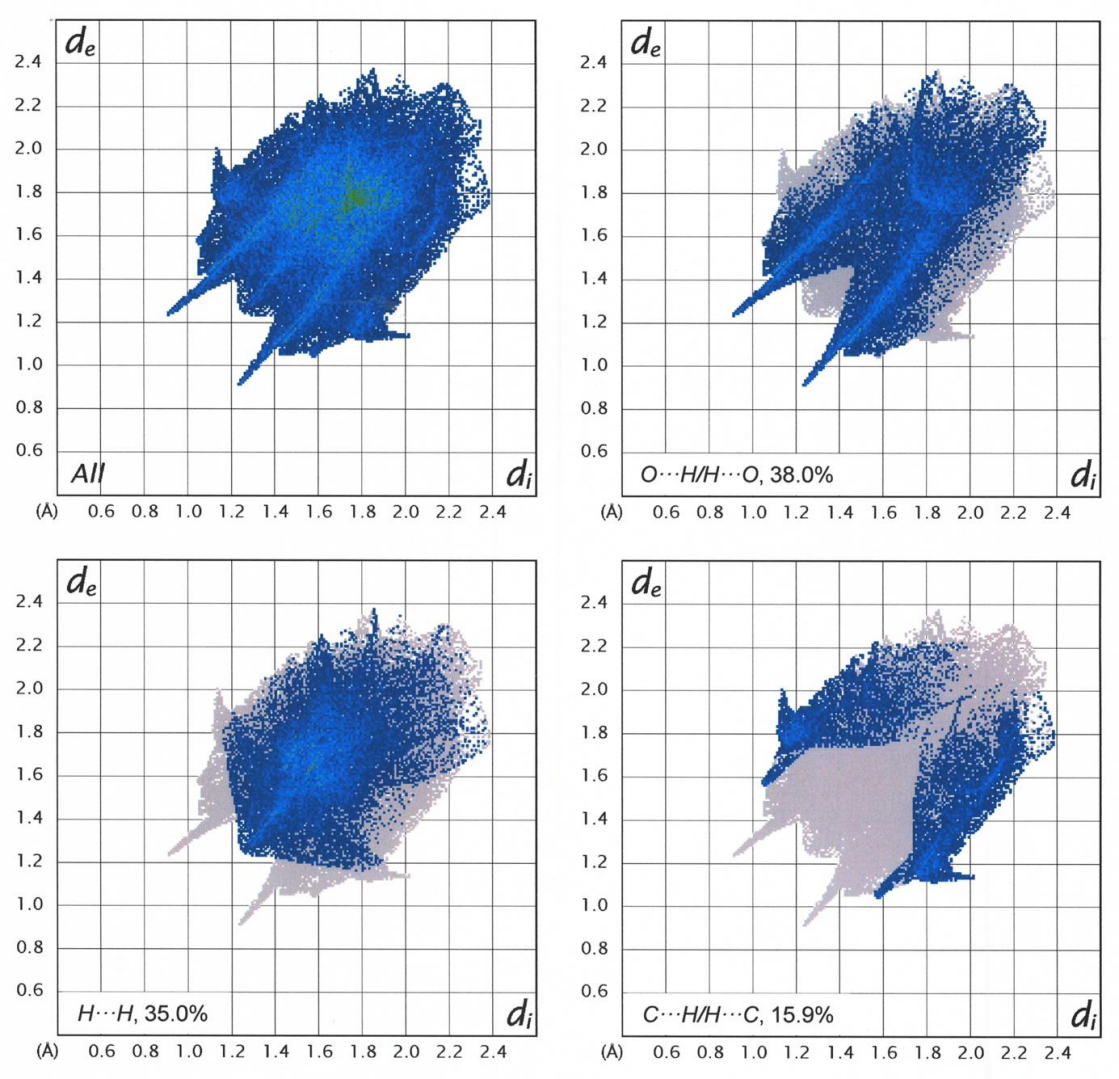
Fingerprint plot for ethyl 2-[(2-oxo-2*H*-chromen-6-yl)­oxy]acetate with all inter­molecular contacts presented in the upper left panel and the O⋯H/H⋯O, C⋯H/H⋯C, and H⋯H contacts illustrated in separate panels (clockwise, respectively).

A Hirshfeld surface, generated by use of *CrystalExplorer* 21.5 (Spackman *et al.*, 2021 ▸) for ethyl 2-[(2-oxo-2*H*-chromen-6-yl)­oxy]acetate is presented in Fig. 6 ▸ with a normalized contact distance (*d*_norm_) set between −0.3446 and 1.3365. Adjacent mol­ecules, both within the plane and above the plane of that depicted with the Hirshfeld surface, are shown along with close C—H⋯O contacts. The C—H⋯O hydrogen bonds that are separately illustrated in Figs. 2 ▸, 3 ▸ and 5 ▸ are collectively shown in Fig. 6 ▸ and emphasize the packing efficiency enabled by the abundance of such juxtapositions. Fig. 7 ▸ illustrates a fingerprint plot with all inter­molecular contacts presented in the upper left panel and the O⋯H/H⋯O, C⋯H/H⋯C, and H⋯H contacts parsed into separate panels (clockwise, respectively). Of these contacts, the O⋯H/H⋯O contribute most importantly to the packing energetics, both because they represent the greatest percentage of the total and because they account for the closest inter­molecular contacts. The distinctive blue fingers observed in the *d*_e_ + *d*_i_ ≃ 2.2–2.6 territory of Fig. 7 ▸, upper right, have their origin in these non-classical C—H⋯O hydrogen bonds.

## 3. Database survey

A variety of chromen-2-ones that are substituted in the 6-position of the ring system have been characterized structurally by X-ray diffraction. Examples include 6-meth­oxy­coumarin (Baures *et al.*, 2002 ▸), 6-benzyl­oxycoumarin (Adfa *et al.*, 2010 ▸), 6-acet­oxy­coumarin (Murthy *et al.*, 1988 ▸), 6-(quin­oxalin-2-yl)coumarin (Bandaru *et al.*, 2019 ▸), 6-(4-*tert*- butyl­benzoate)coumarin (Kenfack Tsobnang *et al.*, 2024 ▸), and 6-(2-iodo­phen­oxy)coumarin (Wang *et al.*, 2022 ▸). Of these, only 6-meth­oxy­coumarin has a planar mol­ecular structure and therefore a sheetlike packing arrangement in the crystalline state that is analogous to that observed for ethyl 2-[(2-oxo-2*H*-chromen-6-yl)­oxy]acetate. Because ar­yloxy substituents in the 6-position of the coumarin ring system are typically not oriented to be in the same plane as the coumarin core, a pattern that such derivatives display is packing as centrosymmetric dyads with with parallel coplanar arrangement of the coumarin cores.

## 4. Synthesis and crystallization

Potassium carbonate (0.512 g, 3.70 mmol) was added to a stirred solution of 6-hy­droxy-2*H*-chromen-2-one (0.200 g, 1.233 mmol) in 10 mL of acetone, and stirring was continued for 30 minutes at 298 K. Bromo­ethyl acetate (0.309 g, 1.850 mmol) was added slowly to the reaction mixture, and upon completion, the temperature was elevated to 313 K with stirring for 12 h. The reaction mixture was filtered, and the filtrate was concentrated to dryness under reduced pressure. The resulting crude solid was purified *via* flash chromatography on silica gel with 20:80 ethyl acetate:hexa­nes as the eluting solvent to yield ethyl 2-[(2-oxo-2*H*-chrome-6-yl)­oxy]acetate as a white solid, m.p. 377–380 K. ^1^H NMR [300 MHz, δ (ppm, in CDCl_3_)]: 7.53 (*d*, *J* = 8.0 Hz, 1 H), 7.21 (*d*, *J* = 8.0 Hz, 1 H), 7.14–7.10 (*m*, 1 H), 6.93 (*d*, *J* = 6.9 Hz, 1 H), 6.39 (*d*, *J* = 7.0 Hz, 1 H), 4.64 (*s*, 2 H), 4.28 (*q*, *J* = 7.4 Hz, 2 H), 1.28 (*t, J* = 7.2 Hz, 3 H). ^13^C NMR (75 MHz, δ (ppm, in CDCl_3_)): 168.4, 160.7, 154.3, 149.0, 143.0, 119.8, 119.2, 117.9, 117.2, 111.6, 65.9, 61.5, 14.1. Diffraction-quality white needle-shaped crystals were obtained by slow cooling of a warm solution of the product in 2:1 ethyl acetate:hexa­nes.

## 5. Refinement

Crystal data, data collection and structure refinement details are summarized in Table 2 ▸. All hydrogen atoms were refined isotropically with displacement parameters 1.2–1.5 times those of the carbon atoms to which they are attached.

## Supplementary Material

Crystal structure: contains datablock(s) I, global. DOI: 10.1107/S2056989024004729/jy2047sup1.cif

Structure factors: contains datablock(s) I. DOI: 10.1107/S2056989024004729/jy2047Isup2.hkl

Supporting information file. DOI: 10.1107/S2056989024004729/jy2047Isup3.cml

CCDC reference: 2356747

Additional supporting information:  crystallographic information; 3D view; checkCIF report

## Figures and Tables

**Table 1:** Hydrogen-bond geometry (Å, °)

*D*—H⋯*A*	*D*—H	H⋯*A*	*D*⋯*A*	*D*—H⋯*A*
C5—H5⋯O4^i^	0.95	2.47	3.1973 (12)	133
C6—H6⋯O3^i^	0.95	2.65	3.5965 (11)	172
C1—H1⋯O1^ii^	0.95	2.61	3.3951 (15)	140
C8—H8⋯O1^ii^	0.95	2.27	3.1536 (12)	154
C12—H12*A*⋯O3^iii^	0.99	2.57	3.5156 (13)	159

Symmetry codes: (i) 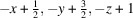 ; (ii) 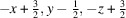 ; (iii) 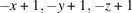 .

**Table 2:** Experimental details

Crystal data	
Chemical formula	C_13_H_12_O_5_
*M*_r_	248.23
Crystal system, space group	Monoclinic, *C*2/*c*
Temperature (K)	150
*a*, *b*, *c* (Å)	8.2188 (4), 13.8709 (7), 20.8370 (11)
β (°)	96.062 (2)
*V* (Å^3^)	2362.2 (2)
*Z*	8
Radiation type	Mo *K*α
μ (mm^−1^)	0.11
Crystal size (mm)	0.22 × 0.11 × 0.07

Data collection	
Diffractometer	Bruker D8 QUEST PHOTON 3 diffractometer
Absorption correction	Numerical (*SADABS*; Krause et al., 2015 ▸)
*T*_min_, *T*_max_	0.95, 0.99
No. of measured, independent and observed [*I* > 2σ(*I*)] reflections	30310, 3031, 2517
*R*_int_	0.031
(sin θ/λ)_max_ (Å^−1^)	0.677

Refinement	
*R*[*F* ^2^ > 2σ(*F* ^2^)], *wR*(*F* ^2^), *S*	0.038, 0.115, 1.08
No. of reflections	3031
No. of parameters	164
H-atom treatment	H-atom parameters constrained
Δρ_max_, Δρ_min_ (e Å^−3^)	0.30, −0.18

Computer programs: *APEX4* and *SAINT* (Bruker, 2021 ▸), *SHELXT/5* (Sheldrick, 2015*a* ▸), *SHELXL2018/3* (Sheldrick, 2015*b* ▸) and *SHELXTL* (Sheldrick, 2008 ▸).

## supplementary crystallographic information

### Crystal data

**Table:**

C_13_H_12_O_5_	*F*(000) = 1040
*M**_r_* = 248.23	*D*_x_ = 1.396 Mg m^−^^3^
Monoclinic, *C*2/*c*	Mo *K*α radiation, λ = 0.71073 Å
*a* = 8.2188 (4) Å	Cell parameters from 9847 reflections
*b* = 13.8709 (7) Å	θ = 2.9–28.7°
*c* = 20.8370 (11) Å	µ = 0.11 mm^−^^1^
β = 96.062 (2)°	*T* = 150 K
*V* = 2362.2 (2) Å^3^	Block, clear colourless
*Z* = 8	0.22 × 0.11 × 0.07 mm

### Data collection

**Table:**

Bruker D8 QUEST PHOTON 3 diffractometer	3031 independent reflections
Radiation source: fine-focus sealed tube	2517 reflections with *I* > 2σ(*I*)
Graphite monochromator	*R*_int_ = 0.031
Detector resolution: 7.3910 pixels mm^-1^	θ_max_ = 28.8°, θ_min_ = 2.9°
φ and ω scans	*h* = −11→11
Absorption correction: numerical (*SADABS*; Krause et al., 2015)	*k* = −18→18
*T*_min_ = 0.95, *T*_max_ = 0.99	*l* = −28→28
30310 measured reflections	

### Refinement

**Table:**

Refinement on *F*^2^	Primary atom site location: dual
Least-squares matrix: full	Secondary atom site location: difference Fourier map
*R*[*F*^2^ > 2σ(*F*^2^)] = 0.038	Hydrogen site location: inferred from neighbouring sites
*wR*(*F*^2^) = 0.115	H-atom parameters constrained
*S* = 1.08	*w* = 1/[σ^2^(*F*_o_^2^) + (0.0593*P*)^2^ + 0.7919*P*] where *P* = (*F*_o_^2^ + 2*F*_c_^2^)/3
3031 reflections	(Δ/σ)_max_ = 0.001
164 parameters	Δρ_max_ = 0.30 e Å^−^^3^
0 restraints	Δρ_min_ = −0.18 e Å^−^^3^

### Special details

**Table:**

Experimental. The diffraction data were obtained from 6 sets of frames, each of width 0.50 ° in ω or φ, collected with scan parameters determined by the "strategy" routine in *APEX4*. The scan time was 10.00 sec/frame.
Geometry. All esds (except the esd in the dihedral angle between two l.s. planes) are estimated using the full covariance matrix. The cell esds are taken into account individually in the estimation of esds in distances, angles and torsion angles; correlations between esds in cell parameters are only used when they are defined by crystal symmetry. An approximate (isotropic) treatment of cell esds is used for estimating esds involving l.s. planes.
Refinement. Refinement of F^2^ against ALL reflections. The weighted R-factor wR and goodness of fit S are based on F^2^, conventional R-factors R are based on F, with F set to zero for negative F^2^. The threshold expression of F^2^ > 2sigma(F^2^) is used only for calculating R-factors(gt) etc. and is not relevant to the choice of reflections for refinement. R-factors based on F^2^ are statistically about twice as large as those based on F, and R- factors based on ALL data will be even larger.

### Fractional atomic coordinates and isotropic or equivalent isotropic displacement parameters (Å^2^)

**Table:**

	*x*	*y*	*z*	*U*_iso_*/*U*_eq_	
O1	0.75633 (12)	1.09654 (7)	0.74589 (4)	0.0550 (3)	
O2	0.61257 (9)	1.00599 (5)	0.67405 (3)	0.0340 (2)	
O3	0.39172 (8)	0.65701 (5)	0.57181 (3)	0.02737 (17)	
O4	0.30429 (9)	0.48767 (5)	0.51199 (3)	0.03335 (19)	
O5	0.43669 (8)	0.40506 (5)	0.59491 (3)	0.02889 (18)	
C1	0.72524 (12)	0.84028 (8)	0.74031 (5)	0.0296 (2)	
H1	0.764445	0.783825	0.762671	0.036*	
C2	0.77358 (13)	0.92769 (8)	0.76270 (5)	0.0341 (2)	
H2	0.846404	0.932070	0.801150	0.041*	
C3	0.71754 (14)	1.01526 (8)	0.72973 (5)	0.0366 (3)	
C4	0.56065 (11)	0.91657 (7)	0.65061 (4)	0.0261 (2)	
C5	0.45308 (12)	0.91484 (7)	0.59435 (5)	0.0288 (2)	
H5	0.417946	0.973141	0.573336	0.035*	
C6	0.39835 (11)	0.82698 (7)	0.56963 (4)	0.0268 (2)	
H6	0.324788	0.824656	0.531298	0.032*	
C7	0.45074 (11)	0.74109 (7)	0.60079 (4)	0.0238 (2)	
C8	0.55704 (11)	0.74335 (7)	0.65691 (4)	0.0246 (2)	
H8	0.591389	0.685046	0.678111	0.030*	
C9	0.61368 (11)	0.83234 (7)	0.68224 (4)	0.0246 (2)	
C10	0.45751 (12)	0.57113 (7)	0.60081 (4)	0.0258 (2)	
H10A	0.430090	0.566985	0.645869	0.031*	
H10B	0.578154	0.571536	0.601579	0.031*	
C11	0.38763 (11)	0.48537 (7)	0.56293 (4)	0.0249 (2)	
C12	0.38869 (13)	0.31449 (7)	0.56288 (5)	0.0316 (2)	
H12A	0.426499	0.312769	0.519332	0.038*	
H12B	0.268095	0.307832	0.558270	0.038*	
C13	0.46617 (15)	0.23374 (8)	0.60369 (6)	0.0411 (3)	
H13A	0.434714	0.171748	0.583483	0.062*	
H13B	0.428707	0.236471	0.646769	0.062*	
H13C	0.585489	0.240449	0.607364	0.062*	

### Atomic displacement parameters (Å^2^)

**Table:**

	*U*^11^	*U*^22^	*U*^33^	*U*^12^	*U*^13^	*U*^23^
O1	0.0740 (6)	0.0406 (5)	0.0461 (5)	−0.0193 (4)	−0.0137 (4)	−0.0108 (4)
O2	0.0400 (4)	0.0273 (4)	0.0323 (4)	−0.0061 (3)	−0.0069 (3)	−0.0044 (3)
O3	0.0308 (4)	0.0226 (3)	0.0261 (3)	−0.0025 (2)	−0.0093 (3)	0.0009 (2)
O4	0.0369 (4)	0.0320 (4)	0.0283 (4)	−0.0025 (3)	−0.0101 (3)	0.0005 (3)
O5	0.0350 (4)	0.0239 (3)	0.0263 (3)	−0.0031 (3)	−0.0039 (3)	0.0020 (3)
C1	0.0275 (5)	0.0371 (5)	0.0229 (4)	−0.0028 (4)	−0.0034 (4)	0.0001 (4)
C2	0.0325 (5)	0.0440 (6)	0.0242 (4)	−0.0084 (4)	−0.0044 (4)	−0.0043 (4)
C3	0.0395 (6)	0.0400 (6)	0.0291 (5)	−0.0112 (4)	−0.0029 (4)	−0.0079 (4)
C4	0.0261 (4)	0.0259 (5)	0.0254 (4)	−0.0044 (3)	−0.0014 (3)	−0.0038 (3)
C5	0.0306 (5)	0.0256 (5)	0.0285 (5)	0.0000 (4)	−0.0059 (4)	0.0020 (4)
C6	0.0263 (4)	0.0277 (5)	0.0245 (4)	−0.0011 (3)	−0.0071 (3)	0.0017 (3)
C7	0.0226 (4)	0.0244 (4)	0.0235 (4)	−0.0025 (3)	−0.0024 (3)	−0.0005 (3)
C8	0.0244 (4)	0.0265 (4)	0.0218 (4)	−0.0006 (3)	−0.0029 (3)	0.0027 (3)
C9	0.0220 (4)	0.0309 (5)	0.0203 (4)	−0.0022 (3)	−0.0015 (3)	−0.0009 (3)
C10	0.0288 (5)	0.0245 (4)	0.0226 (4)	−0.0024 (3)	−0.0043 (3)	0.0025 (3)
C11	0.0251 (4)	0.0257 (4)	0.0233 (4)	−0.0024 (3)	−0.0001 (3)	0.0022 (3)
C12	0.0384 (5)	0.0247 (5)	0.0317 (5)	−0.0034 (4)	0.0036 (4)	−0.0026 (4)
C13	0.0418 (6)	0.0289 (5)	0.0534 (7)	0.0043 (4)	0.0082 (5)	0.0041 (5)

### Geometric parameters (Å, º)

**Table:**

O1—C3	1.2096 (13)	C5—H5	0.9500
O2—C3	1.3769 (12)	C6—C7	1.4026 (13)
O2—C4	1.3838 (11)	C6—H6	0.9500
O3—C7	1.3778 (11)	C7—C8	1.3842 (12)
O3—C10	1.4169 (11)	C8—C9	1.4025 (13)
O4—C11	1.2013 (11)	C8—H8	0.9500
O5—C11	1.3383 (11)	C10—C11	1.5074 (12)
O5—C12	1.4573 (11)	C10—H10A	0.9900
C1—C2	1.3440 (14)	C10—H10B	0.9900
C1—C9	1.4432 (12)	C12—C13	1.5062 (15)
C1—H1	0.9500	C12—H12A	0.9900
C2—C3	1.4467 (16)	C12—H12B	0.9900
C2—H2	0.9500	C13—H13A	0.9800
C4—C9	1.3888 (13)	C13—H13B	0.9800
C4—C5	1.3920 (12)	C13—H13C	0.9800
C5—C6	1.3798 (13)		
			
C3—O2—C4	121.57 (8)	C9—C8—H8	120.2
C7—O3—C10	115.08 (7)	C4—C9—C8	119.14 (8)
C11—O5—C12	115.90 (7)	C4—C9—C1	118.25 (9)
C2—C1—C9	119.89 (9)	C8—C9—C1	122.61 (9)
C2—C1—H1	120.1	O3—C10—C11	109.44 (7)
C9—C1—H1	120.1	O3—C10—H10A	109.8
C1—C2—C3	121.67 (8)	C11—C10—H10A	109.8
C1—C2—H2	119.2	O3—C10—H10B	109.8
C3—C2—H2	119.2	C11—C10—H10B	109.8
O1—C3—O2	116.42 (10)	H10A—C10—H10B	108.2
O1—C3—C2	126.09 (10)	O4—C11—O5	125.15 (9)
O2—C3—C2	117.48 (9)	O4—C11—C10	126.30 (9)
O2—C4—C9	121.13 (8)	O5—C11—C10	108.54 (7)
O2—C4—C5	117.22 (9)	O5—C12—C13	107.74 (8)
C9—C4—C5	121.64 (9)	O5—C12—H12A	110.2
C6—C5—C4	118.87 (9)	C13—C12—H12A	110.2
C6—C5—H5	120.6	O5—C12—H12B	110.2
C4—C5—H5	120.6	C13—C12—H12B	110.2
C5—C6—C7	120.35 (8)	H12A—C12—H12B	108.5
C5—C6—H6	119.8	C12—C13—H13A	109.5
C7—C6—H6	119.8	C12—C13—H13B	109.5
O3—C7—C8	123.44 (8)	H13A—C13—H13B	109.5
O3—C7—C6	116.07 (7)	C12—C13—H13C	109.5
C8—C7—C6	120.49 (8)	H13A—C13—H13C	109.5
C7—C8—C9	119.51 (9)	H13B—C13—H13C	109.5
C7—C8—H8	120.2		
			
C9—C1—C2—C3	−0.47 (16)	C6—C7—C8—C9	−0.63 (14)
C4—O2—C3—O1	179.55 (10)	O2—C4—C9—C8	179.62 (8)
C4—O2—C3—C2	0.72 (15)	C5—C4—C9—C8	−0.16 (15)
C1—C2—C3—O1	−178.93 (12)	O2—C4—C9—C1	−0.21 (14)
C1—C2—C3—O2	−0.23 (16)	C5—C4—C9—C1	180.00 (8)
C3—O2—C4—C9	−0.50 (15)	C7—C8—C9—C4	0.50 (14)
C3—O2—C4—C5	179.29 (9)	C7—C8—C9—C1	−179.67 (8)
O2—C4—C5—C6	−179.84 (9)	C2—C1—C9—C4	0.69 (14)
C9—C4—C5—C6	−0.05 (15)	C2—C1—C9—C8	−179.14 (9)
C4—C5—C6—C7	−0.08 (15)	C7—O3—C10—C11	−177.82 (7)
C10—O3—C7—C8	−4.68 (13)	C12—O5—C11—O4	2.60 (14)
C10—O3—C7—C6	174.59 (8)	C12—O5—C11—C10	−176.17 (7)
C5—C6—C7—O3	−178.87 (8)	O3—C10—C11—O4	8.06 (14)
C5—C6—C7—C8	0.43 (15)	O3—C10—C11—O5	−173.20 (7)
O3—C7—C8—C9	178.61 (8)	C11—O5—C12—C13	175.57 (8)

### Hydrogen-bond geometry (Å, º)

**Table:**

*D*—H···*A*	*D*—H	H···*A*	*D*···*A*	*D*—H···*A*
C5—H5···O4^i^	0.95	2.47	3.1973 (12)	133
C6—H6···O3^i^	0.95	2.65	3.5965 (11)	172
C1—H1···O1^ii^	0.95	2.61	3.3951 (15)	140
C8—H8···O1^ii^	0.95	2.27	3.1536 (12)	154
C12—H12*A*···O3^iii^	0.99	2.57	3.5156 (13)	159

Symmetry codes: (i) −*x*+1/2, −*y*+3/2, −*z*+1; (ii) −*x*+3/2, *y*−1/2, −*z*+3/2; (iii) −*x*+1, −*y*+1, −*z*+1.
